# The Variable Effects of NSAIDs on Osteotomy Healing and Opioid Consumption

**DOI:** 10.5435/JAAOSGlobal-D-20-00039

**Published:** 2020-04-06

**Authors:** Austin Fragomen, Jaehee Suh, Kelsey Matta, Thomas H. McCoy, Kamber L. Hart, S. Robert Rozbruch

**Affiliations:** From the Department of Clinical Orthopaedics, Weill Medical College of Cornell University, The Hospital for Special Surgery (Dr. Fragomen and Dr. Rozbruch); Limb Lengthening and Complex Reconstruction Service, The Hospital for Special Surgery (Dr. Fragomen and Dr. Rozbruch); Hospital for Special Surgery (Ms. Suh), New York, NY; Geisinger Commonwealth School of Medicine, Scranton, PA (Ms. Matta); Department of Medicine and Psychiatry, Harvard Medical School, Center for Quantitative Health, Simches Research Building, Massachusetts General Hospital and Harvard Medical School (Dr. McCoy); and Center for Quantitative Health, Simches Research Building, Massachusetts General Hospital and Harvard Medical School (Ms. Hart), Boston, MA.

## Abstract

In an effort to fight the opioid epidemic, an NSAID pain protocol was created for osteotomy patients. The study asked if NSAIDs negatively affect bone healing or reduce the need for opioids.

**Methods:**

This was a retrospective review of 155 limbs that underwent osteotomy of a long bone with fixation. Patients received an NSAID-free protocol or an NSAID protocol. Time to union and bone healing index were recorded.

**Results:**

There was not a significant difference in the time to union (*P* = 0.89) or bone healing index (*P* = 0.07). In the deformity correction group, the total milligrams of morphine equivalents prescribed after discharge was significantly less in patients receiving NSAIDs (*P* < 0.001).

**Conclusions:**

The use of NSAIDs after osteotomy surgery did not negatively affect bone healing and resulted in a dramatic decrease in narcotic consumption for deformity correction patients.

**Level of Evidence:**

Level III retrospective cohort study

The role of prescription medications in the opioid epidemic has been pushed to center stage by lawmakers and the media in North America.^1^ Orthopaedic surgery has been identified as a top prescribing field, and implicated in overprescribing, of narcotic analgesic medication.^2,3^ New persistent opioid use after common orthopaedic procedures occurs with an incidence of 4.3% to 8.2%.^2,4^ Although predisposing patient factors (including anxiety, catastrophic thinking, and preoperative narcotic use) have been recognized to increase the risk of addiction,^1^ a reduction in opioid prescribing needs to be the primary means of controlling this issue. Orthopaedic centers have responded by developing opioid-prescribing guidelines for individual orthopaedic procedures in an effort to reduce the distribution of excess pills.^5^ However, reducing the quantity of narcotics dispensed does not obviate the need for additional nonopioid-based pain medications. Multimodal analgesia, including NSAIDs, has displayed synergistic, beneficial effects on pain control,^6,7^ but a fear that NSAIDs would inhibit bone healing made surgeons reluctant to use them after osteotomy surgery.^8^ Internal quality control and a dedication to systems-based learning inspired our own institute to reanalyze our prescribing practices for patients undergoing limb lengthening and deformity correction surgery where opioids had been the cornerstone for controlling pain secondary to soft-tissue stretching. An analysis of the literature on NSAID use and fracture healing revealed much contradictory^8,9^ or inconclusive data.^10^ Some studies even suggested that *opioid* use, rather than NSAID use, after trauma markedly increased the risk of fracture nonunion.^11^ In a collaboration with our anesthesia team, a new postoperative multimodal pain protocol emphasizing NSAID administration was created for all osteotomy patients. The “new protocol” included intravenous (IV) ketorolac and IV acetaminophen for the first day after surgery, followed by oral celecoxib or meloxicam and oral acetaminophen. Patients were noted to have less pain in the entire postoperative period that was attributed to the NSAID and acetaminophen's ability to control pain but was just as likely secondary to the removal of narcotics which create habituation and hyperalgesia, actually increasing the baseline pain over time. This study looks back at patients who underwent surgery under the older protocol (NSAID-free protocol), which relied primarily on narcotics for pain control, and compares them with the new protocol that incorporates standing NSAIDs and acetaminophen into the postoperative pain regimen. The following questions were asked: (1) Did the use of NSAIDs negatively affect osteotomy healing compared with no NSAIDs? (2) Did the use of NSAIDs reduce the need for opioids compared with no NSAIDs? (3) Among the patients taking NSAIDs, did the quantity of NSAIDs and narcotics consumed differ between patients undergoing osteotomy for deformity correction or osteotomy for bone lengthening?

## Methods

This was a retrospective review of a consecutive series of 155 limbs in 136 patients treated at a single center by either of the two orthopaedic surgeons. All patients underwent osteotomy of a long bone with fixation. Osteotomy was performed by one of the two methods: (1) for lengthening cases and deformity cases fixed with an intramedullary (IM) nail or an external fixator (frame), the osteotomy was made percutaneously by creating multiple drill holes and then cracking the bone with a corticotomy osteotome; (2) for opening-wedge high tibial and distal femoral osteotomies, the bone cut was done using a saw, grafted with freeze-dried allograft, and fixed with a plate. All patients received spinal epidural anesthesia during surgery. In femur osteotomy patients, the epidural catheter was used overnight to control pain through a patient-controlled anesthesia pump. In tibia osteotomy patients, where compartment syndrome is a concern, IV patient-controlled anesthesia allowed for uninhibited neurologic monitoring and was used for pain control for the first night. All patients were written for oral narcotics, either oxycodone or hydromorphone, immediately after surgery, and this medication was continued postoperatively as needed for pain control.

Patients receiving the NSAID-free protocol were not allowed to have NSAIDs at any point during or after surgery. Intraoperative ketorolac was forbidden, and its omission was a part of the anesthesia protocol. Patients were counseled during the routine preoperative visit to avoid NSAIDs. The fear that NSAIDs would slow healing was also spelled out in a patient handout that all preoperative patients received. Patients were instructed routinely during postoperative visits to not take NSAIDs because this was the routine practice at that time. Patients were allowed to take acetaminophen, but this was not reinforced. Oral narcotics were used as the mainstay of pain control. Patients were discharged with narcotic pain medication, and refills were provided as needed.

Patients receiving the NSAID protocol were started on ketorolac 15 mg IV intraoperatively and then postoperatively (op) every 6 hours for three additional doses. IV acetaminophen was started intraoperatively and continued post-op every 6 hours for three additional doses. After these medications expired on post-op day 1, patients were given either celecoxib 200 mg twice daily or meloxicam 15 mg once daily as a standing order. The decision of which NSAID to use was based on chronology; celecoxib was used at first, but increasing difficulty with obtaining preauthorization drove us to use meloxicam, which did not require insurance authorization. They also received acetaminophen 1000 mg orally every 6 hours as a standing order (Table 1). Patients were discharged with the same NSAID they received during hospitalization and acetaminophen as well as a narcotic prescription. Patients were taught to manage pain by first taking the acetaminophen, then if needed to add the NSAID, and then if needed to add the opioid. These teaching sessions occurred during the preoperative visit with our trained registered nurse, the discharge medication reconciliation with our trained physician assistant, and during early postoperative visits with the surgeons.

**Table 1:** NSAID Protocol

Medication	POD 0	POD 1	POD 2 and beyond
Ketorolac 15 mg	Q6 hr 4 doses	—	—
IV acetaminophen 1,000 mg	Q6 hr 4 doses		
IV PCA (morphine/hydromorphone) or epidural PCA	Overnight	—	—
Oxycodone 5–15 mg	Q3 hr PRN	Q3 hr PRN	Q3 hr PRN
Meloxicam 15 mg or celecoxib 200 mg	—	QD, Q12 hr	QD, Q12 hr
PO acetaminophen 1,000 mg	—	Q6 hr	Q6 hr

IV = intravenous, PCA = patient-controlled anesthesia, PO = oral, POD = post operative day

Follow-up schedules were based on the rate of correction of osteotomy patients: acute deformity correction patients were seen monthly in the office for examination and radiographs. Gradual deformity correction and lengthening patients were seen every 2 weeks. All patients received thrombosis prophylaxis with either rivaroxaban, enoxaparin, or aspirin for 2 weeks postsurgery. All NSAID protocol patients were prescribed pantoprazole to prevent bleeding ulcers. Weight-bearing allowance was based on surgical treatment.

## Research Method

This retrospective cohort study reviewed a consecutive series of osteotomies of 155 limbs in 136 patients. Nineteen patients underwent bilateral osteotomy that occurred in a staged fashion and were counted as separate limbs. Simultaneous bilateral osteotomies were counted as one limb in this study; the time to union was averaged between the two limbs. The entire cohort straddled a major change in practice: the adaptation of NSAIDs into the post op pain protocol. This change was implemented on August 1, 2016. Every patient who underwent osteotomy of the lower extremity for deformity correction and/or limb lengthening between February 2016 and March 2017 was included. The study start date was selected because this was the date of implementation of the hospital-wide electronic medical record (EMR) system with e-prescribing, allowing easy access to all aspects of patient care documentation. The NSAID-free protocol spanned for 6 months from February 1, 2016, to July 31, 2016. The NSAID protocol sought to span a similar 6-month period from September 1, 2016, to March 1, 2017. Patients treated during the transition in August would be included in the appropriate group based on which protocol they received during the transition or eliminated if they received a mixed protocol. Patients were treated with a combination of internal and external implants. Patients treated with carbon fiber-reinforced polymer implants were excluded because of their high propensity for delayed bone consolidation.^12^ Data collection started in June 2018. Demographics, time to union, and incidence of nonunion were recorded based on the review of charts and radiographs. The date of union was clearly documented in the charts by the surgeon in nearly all cases. If the data collectors were not sure of a date of union, then the radiology reports of the radiographs were used to determine union. Total medication administered during and after surgery for the entire post-op period was recorded from chart review. During the hospital admission, the quantity of medication administered was very well documented. In the outpatient setting, all prescriptions were e-prescribed through the EMR, except acetaminophen, which was mostly purchased over the counter. Post-op medications could only be tracked through the EMR by the number of pills prescribed with each refill. The date that the refill was issued and the quantity of pills were well documented. For narcotics, a 2-week supply was issued. For NSAIDs, a 1-month supply was issued. There was no way of knowing if patients actually took the medication. The study assumed that patients took all of the medication prescribed until they ran out. Patients who required a refill can safely be assumed to have taken all of their medication before the refill request. Acetaminophen was not tracked because it was not prescribed through the system. Patients were divided into two osteotomy groups. Those undergoing deformity correction with less than 2 cm of lengthening performed with any technique were considered the deformity correction cohort. Those patients who underwent lengthening of greater than 2 cm with or without a concomitant deformity correction were considered the lengthening cohort.

Conservation of bony union was the greatest clinical concern and was the primary outcome for this study. Time to union, a reliable indicator of osteogenic suppression, was recorded for all deformity osteotomy patients, and bone healing index (BHI) (BHI = days until union/cm lengthening) was calculated for all patients undergoing limb lengthening. BHI does vary based on the amount of lengthening performed with greater lengthening producing lower BHI ratios. A bone was declared “united” when three of four cortices were visibly bridged on radiographs. For all patients, nonunion status was defined as a bone that failed to heal after surgery and required further surgical intervention to unite. For deformity correction patients, we used the standard Kaplan-Meier analysis to compare time to union between protocols. Then, because the covariates met the assumptions of proportional hazards, we used a Cox proportional hazard model to compare the union between protocols including age, sex, laterality, and smoking as covariates.

Patients who underwent limb lengthening were analyzed separately because time to union was a product of the distance the bone was lengthened. The number of patients undergoing distraction osteogenesis was markedly higher in the NSAID-free protocol group, and therefore, additional limb lengthening patients were added to the NSAID protocol group by expanding the study period from March 2017 to September 2017 exclusively for patients who underwent distraction osteogenesis for lengthening greater than 2 cm. This added 12 consecutive patients (16 limbs) for analysis. Distraction osteogenesis was thought to be a particularly sensitive indicator for any variable that might affect bone healing, thus warranting the addition of patients to improve study power. BHI was compared between the two protocols using the Wilcoxon-Mann-Whitney test. The relationship between the length of time taking NSAIDs or narcotics and BHI was assessed using the Spearman rank correlation.

For both the deformity correction and lengthening cohorts, the total milligrams of morphine equivalents provided after discharge were compared between the NSAID-free and NSAID protocols using a Wilcoxon-Mann-Whitney test. In addition, the number of narcotic pills dispensed during inpatient treatment was compared between the NSAID-free and NSAID protocols using a Wilcoxon-Mann-Whitney test for each cohort. For patients in the NSAID protocol, the number of days on NSAIDs and days on narcotics was compared between deformity and lengthening patients using a Wilcoxon-Mann-Whitney test (the number of days taking narcotics and NSAIDs was estimated by assuming that the patients followed the sig on the prescription). Finally, the milligrams of morphine equivalents prescribed were compared between deformity and lengthening patients receiving the NSAID protocol using a Wilcoxon-Mann-Whitney test.

## Results

There were 170 participants eligible for the study. Fifteen NSAID protocol and 14 NSAID-free protocol patients had undergone fixation of their osteotomy with carbon fiber-reinforced polymer implants and were eliminated from the study, leaving 141 patients. Five patients in the NSAID-free protocol were found to have been prescribed NSAIDs in the post-op period while transitioning to the NSAID protocol. These patients were removed from the study, leaving 155 limbs in 136 patients for analysis. The median follow-up time was 893 days (29.3 months) (131.81 days interquartile range). The minimum follow-up was 497 days (16.3 months).

**Figure 1:**
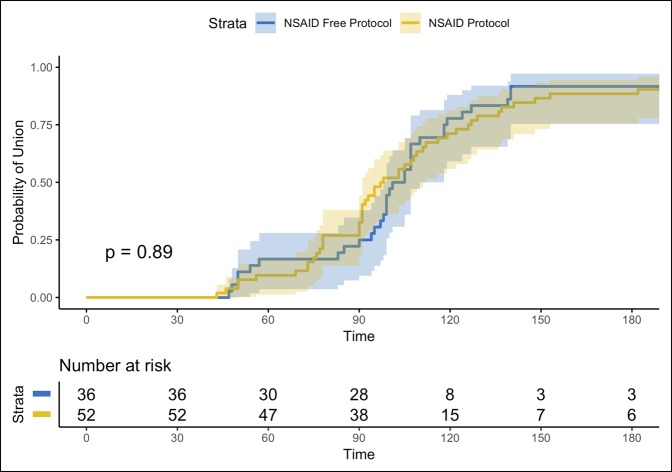
*P* = 0.89; Chart showing the time to union is plotted for deformity patients in each protocol using a Kaplan-Meier method, where union = failure and nonunion = survival. Both protocols demonstrate similar patterns of healing and nonunion (>180 days) incidence.

In a cohort of 88 limbs treated for deformity correction, 36 received the NSAID-free protocol and 52 received the NSAID protocol. Three patients with nonunion were identified, two in the NSAID group and one in the NSAID-free group. In crude analysis, there was not a significant difference between protocols in the time to union (103 days: NSAID-free, 97.5 days: NSAID) (Kaplan-Meier log rank *P* = 0.89) (Figure 1) or in the hazard ratio for union after adjustment for age, sex, laterality, and smoking (hazard ratio: 0.90, 95% CI: 0.54 to 1.49) (Tables 2 and 3). Concern that the higher number of young people (with presumed faster healing) in the NSAID protocol deformity correction cohort might skew the data led to additional analysis that excluded children aged 16 and younger. The results were not qualitatively different, excluding these individuals (Table 4).

**Table 2:** Demographic and Clinical Data for Deformity Correction Patients (Number of Limbs = 88)

Variable	NSAID-Free Protocol, N = 36	NSAID Protocol, N = 52	*P* Value	Method
Age (yr), median (IQR)	45 (36.0)	29 (28.8)	0.03^a^	Wilcoxon-Mann-Whitney test
Sex (male), N (%)	18 (50.0)	25 (48.1)	0.99	Fisher exact test
Laterality (right), N (%)	24 (66.7)	24 (46.2)	0.08	Fisher exact test
Children 16 and younger, N (%)	0 (0.0)	5 (9.6)	0.08	Fisher exact test
Smoking, N (%)	1 (2.8)	10 (19.2)	0.02^a^	Fisher exact test
Etiology			0.01^a^	Fisher exact test
Congenital, N (%)	23 (63.9)	46 (88.5)		
Post traumatic, N (%)	12 (33.3)	6 (11.5)		
Bone				
Femur, N (%)	9 (25.0)	19 (36.5)	0.35	Fisher exact test
Tibia, N (%)	27 (75.0)	33 (63.5)	0.35	Fisher exact test
Static IM nail, N (%)	7 (19.5)	13 (25.0)	0.78	Fisher exact test
Titanium plate, N (%)	15 (41.7)	21 (40.4)	1.00	Fisher exact test
External fixator, N (%)	14 (38.9)	18 (34.6)	0.82	Fisher exact test
Results				
Time to union (d), median (IQR)	103 (25.2)	97.5 (48.2)	0.89	Kaplan-Meier log rank test

IQR = interquartile range, IM = Intramedullary.

a statistically significant

**Table 3:** Demographic and Clinical Data for Lengthening Patients (Number of Limbs = 67)

Variable	NSAID-Free Protocol, N = 31	NSAID Protocol, N = 36	*P* Value	Method
Age (yr), median (IQR)	26 (26)	27 (19.2)	0.64	Wilcoxon-Mann-Whitney test
Sex (male), N (%)	21 (67.7)	21 (58.3)	0.46	Fisher exact test
Laterality (right), N (%)	10 (32.3)	17 (47.2)	0.32	Fisher exact test
Children 16 and younger, N (%)	6 (19.4)	8 (22.2)	0.99	Fisher exact test
Smoking, N (%)	4 (12.9)	3 (8.3)	0.70	Fisher exact test
Osteomyelitis, N (%)	2 (6.5)	3 (8.3)	0.99	Fisher exact test
Etiology			1.0	Fisher exact test
Congenital, N (%)	21 (67.7)	24 (66.7)		
Post traumatic, N (%)	10 (32.3)	12 (33.3)		
Bone				
Femur, N (%)	22 (71.0)	24 (66.6)	0.79	Fisher exact test
Tibia, N (%)	9 (29.0)	12 (33.3)	0.79	Fisher exact test
Precise IM nail, N (%)	25 (80.6)	27 (75.0)	0.77	Fisher exact test
External fixator, N (%)	6 (19.4)	9 (25.0)	0.77	Fisher exact test
Lengthening (mm), median (IQR)	42 (29)	36 (32.8)	0.91	Wilcoxon-Mann-Whitney test
BHI (d/cm), median (IQR)	29 (17)	38 (23)	0.07	Wilcoxon-Mann-Whitney test

BHI = bone healing index, IQR = interquartile range, IM = Intramedullary

**Table 4:** Demographic and Clinical Data for Deformity Correction Patients Excluding Children Aged 16 and Younger (Number of Limbs = 83)

Variable	NSAID-Free Protocol, N = 36	NSAID Protocol, N = 47	*P* Value	Method
Age (yr), median (IQR)	45 (36.0)	33 (29)	0.13	Wilcoxon-Mann-Whitney test
Sex (male), N (%)	18 (50.0)	21 (44.7)	0.66	Fisher exact test
Laterality (right), N (%)	24 (66.7)	22 (46.8)	0.08	Fisher exact test
Chronic pain, N (%)	4 (11.1)	1 (2.1)	0.16	Fisher exact test
Smoking, N (%)	1 (2.8)	6 (12.8)	0.13	Fisher exact test
Etiology			0.03^a^	Fisher exact test
Congenital, N (%)	23 (63.9)	41 (87.2)		
Post traumatic, N (%)	12 (33.3)	6 (12.8)		
Bone				
Femur, N (%)	9 (25.0)	16 (34.0)	0.47	Fisher exact test
Tibia, N (%)	27 (75.0)	31 (66.0)	0.47	Fisher exact test
Precise IM nail, N (%)	1 (2.8)	2 (4.3)	1.00	Fisher exact test
Static IM nail, N (%)	6 (16.7)	9 (19.1)	1.00	Fisher exact test
Titanium plate, N (%)	15 (41.7)	20 (42.6)	1.00	Fisher exact test
External fixator, N (%)	14 (38.9)	16 (34.0)	0.65	Fisher exact test
Results				
Time to union (d)	103 (25.2)	98 (50)	0.76	Kaplan-Meier log rank test

IQR = interquartile range, IM = Intramedullary.

a statistically significant

**Figure 2:**
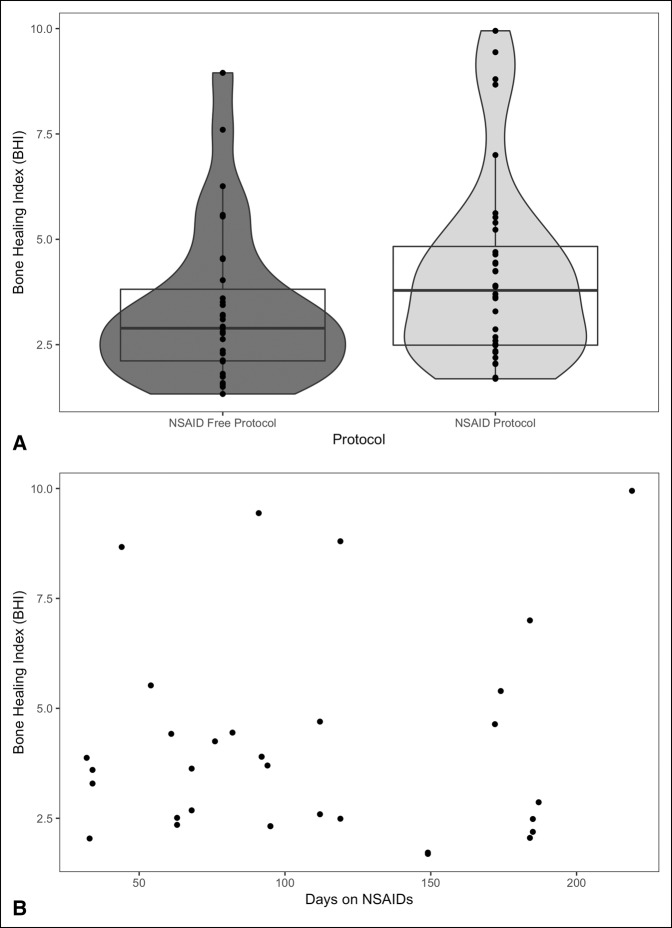
**A**, Chart of the middle line indicating the median; the top and bottom of the box delineate the 25th and 75th percentiles. The shaded areas indicate the distribution and density of the data. The BHI was lower (faster healing) in the NSAID-free protocol patients, *P* = 0.07. **B**, Scatter plot of BHI versus days on NSAIDs showing no correlation, *P* = 0.73. This implies that there is not a trend for NSAIDs to delay healing with longer exposure, which may have been inferred from (**A**). BHI = bone healing index

Among the 67 limbs receiving the lengthening protocol, 31 received the NSAID-free protocol and 36 received the NSAID protocol (Figure 2, A). This study could not show a significant difference in BHI between the groups (*P* = 0.07). The amount of lengthening performed in each cohort was very similar (*P* = 0.91s). Further analysis showed no relationship between length of time taking NSAIDs and prolonged BHI (*P* = 0.73) (Figure 2, B). Similarly, the length of time taking narcotics was not correlated with prolonged BHI (*P* = 0.73).

**Figure 3:**
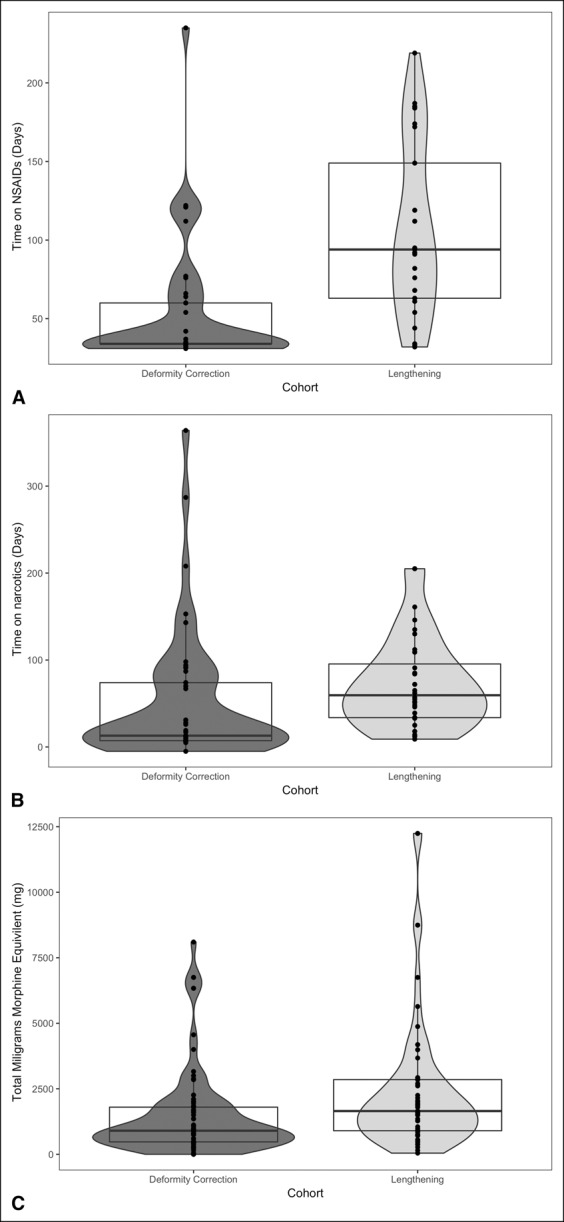
**A**, Among the NSAID protocol patients, those undergoing lengthening surgery were exposed to NSAIDs for significantly longer time than the deformity correction patients, *P* < 0.001. The middle line indicates the median; the top and bottom of the box delineate the 25th and 75th percentiles. The shaded areas indicate the distribution and density of the data. This implies that lengthening produced more sustained pain than deformity correction and drove patients to continue taking NSAIDs. **B**, Among the NSAID protocol patients, those undergoing lengthening surgery were exposed to opioids for significantly longer than the deformity correction patients, *P* < 0.001. The middle line indicates the median; the top and bottom of the box delineate the 25th and 75th percentiles. The shaded areas indicate the distribution and density of the data. The lengthening patients also needed more narcotic for the sustained pain. **C**, Among the NSAID protocol patients, those undergoing lengthening surgery consumed significantly more opioids than those undergoing deformity correction alone, *P* < 0.001. The middle line indicates the median; the top and bottom of the box delineate the 25th and 75th percentiles. The shaded areas indicate the distribution and density of the data. Lengthening patients were on narcotics for a longer time and consumed more narcotics.

In the deformity correction group, the total number of narcotic pills prescribed in the hospital and the total milligrams of morphine equivalents prescribed after discharge were significantly less in patients receiving NSAIDs (<0.001) (Table 5). In patients undergoing lengthening, the total number of narcotic pills prescribed in the hospital and the total milligrams of morphine equivalents prescribed after discharge were not significantly less in patients receiving NSAIDs (*P* = 0.12 and *P* = 0.13, respectively) (Table 6). Among patients receiving the NSAID protocol, lengthening patients had significantly longer NSAID regiments and narcotic regiments after hospital discharge than deformity correction patients (*P* < 0.001; Figure 3, B) and had significantly greater total milligrams of morphine equivalents prescribed than the deformity correction cohort (*P* < 0.001; Figure 3, C).

**Table 5:** Medication Demographics—Deformity Correction Patients

Variable	NSAID Free	NSAID	*P* Value
Inpatient			
Narcotic pills dispensed, median (IQR)	20 (12.5)	12 (11.5)	0.002^a^
Outpatient			
Mg morphine eq (total dose), median (IQR)	1,050 (1,950)	600 (1,204)	<0.001^a^
Days on NSAIDs, median (IQR)	0 (0.0)	34 (26.0)	—

IQR = interquartile range.

a statistically significant.

**Table 6:** Medication Demographics—Lengthening Patients

Variable	NSAID Free	NSAID	*P* Value
Inpatient			
Narcotic pills dispensed, median (IQR)	20 (10.5)	14.5 (12.5)	0.12
Outpatient			
Mg morphine eq (total dose), median (IQR)	1,920 (1,290)	1,425 (1,995)	0.13
Days on NSAIDs, median (IQR)	0 (0.0)	94 (86)	—

IQR = interquartile range

Complications are detailed in Table 7.

**Table 7:** Complications

Complication	NSAID-free Protocol (n)	NSAID Protocol (n)
GI bleed upper	0	1
GI bleed lower	0	1
Gastritis	0	1
Neuritis from lengthening	2	2
VTE	0	1
Equinus contracture postlengthening	0	2
Peroneal nerve	0	1
Nonunion	1	2

GI = gastrointestinal, VTE = venous thromboembolic.

## Discussion

There are many potential causes for delayed and nonunion after osteotomy surgery including patient age, implant choice, smoking, host health, infection, implant loosening, and the use of certain medications including NSAIDs.^13^ Likely, these variables are additive, further complicating the ability to identify a culprit. The need to reduce opioid prescribing in a limb deformity practice inspired the use of NSAIDs and the subsequent study of their impact on bone healing. This investigation, a retrospective glance back at a change in clinical practice, was prone to the pitfalls of level 3 clinical research (namely suboptimal science) yet provides a profound message of hope to the worldwide limb deformity community that is currently avoiding NSAIDs and struggling with opioids. This study showed that the use of NSAIDs had no notable impact on bone healing after osteotomy surgery for lengthening or deformity correction. Using a similar design, Fader et al^14^ retrospectively studied tibial diaphyseal fracture healing after IM nailing in two cohorts: one using opioids and the other using NSAIDs for post-op pain control. NSAID treatment was carried out with normal dosing and for less than 2 weeks duration in most patients enrolled. No notable difference existed in time to union between the treatment groups. Authors concluded that short courses of NSAIDs were safe and effective at controlling postoperative pain after tibia fracture. A recent meta-analysis studied the effect of NSAIDs on long bone healing in fracture, osteotomy, and fusion patients. Authors found that adults were more vulnerable to delayed union when taking NSAIDs. This was particularly true with high doses (ketorolac >120 mg/d) and with prolonged administration of the drug (>2 weeks).^15^ The same study concluded that low dose therapy (ketorolac < 120 mg/d) and short duration of NSAID exposure did not slow healing. In our population of osteotomy patients, NSAID exposure was moderate duration for deformity correction (34 days median) and prolonged duration for osseous lengthening patients (94 days median). The dosing used in our protocol (ketorolac 60 mg/d, celecoxib 400 mg/d, and meloxicam 15 mg/d) would be considered “normal dose” ^16^ or “low dose.”^15^ A meta-analysis of NSAIDs' effect on spinal fusion found that high-dose therapy with ketorolac, even for short courses (<2 weeks), resulted in a high nonunion rate, whereas normal dose courses did not.^16^ The BHI in the patients without NSAID use was lower than that in the patients exposed to prolonged NSAIDs (29 versus 38 days), but the study did not show significance (*P* = 0.07). On further analysis of time on NSAIDs and BHI, we were unable to see any correlation between the duration of NSAID use and the time to bone healing supporting the conclusion that NSAIDs do not slow healing in lengthening surgery. The use of NSAIDs in lengthening patients did not markedly reduce the need for postoperative narcotics as measured by morphine milligram equivalents (MME) dispensed. These findings reinforce the need for other solutions for pain control after lengthening osteotomy surgery.

Complications were infrequent and included gastrointestinal bleeding, gastritis, and venous thromboembolic (VTE) in patients exposed to NSAIDs. The use of concomitant anticoagulants likely contributed to bleeding. Although a proton-pump inhibitor medication was prescribed to mitigate this complication, we do not know if patients took the medicine (Table 7). Gastrointestinal complications did not occur in the NSAID-free cohort, raising the question of what adverse effects are acceptable and further demonstrating the need for a better alternative source of pain control. At the time of latest follow-up, no patient became a new persistent opioid user in either cohort, based on the chart documentation including opioid activity.

This study is limited in its ability to prove that NSAIDs have no effect on osteotomy healing which can only be done through a prospective, randomized controlled trial. We were unable to study the pain level experienced by patients using the NSAID-free and NSAID protocols. Acetaminophen use was an important aspect of the NSAID protocol but was not tracked in the outpatient setting, making it unable to be studied. The number of pills patients consumed was estimated and may have led to an overestimation of treatment duration. The very public opioid crisis may have influenced patients to self-restrict the quantity of narcotic taken, creating a potential bias in narcotic consumption. In addition, patients in the NSAID protocol were counseled to wean off narcotics as soon as possible which could have affected the study. The deformity correction groups were not well matched. The average age in the NSAID-free protocol was markedly greater, and the number of smokers in the NSAID protocol group was markedly higher. The analysis was rerun, excluding patients aged 16 years and younger, which upheld the findings of the original analysis with no notable difference in the time to union with the change to NSAID use. The ability to measure time to union was limited to the patient follow-up intervals (when the radiograph was obtained) which were based on clinical need. These intervals were not completely uniform between patients. The incidence of nonunion, which was a primary outcome for this study, was quite infrequent, making the failure sample size small and the power of the study inadequate to make strong conclusions despite the large number of osteotomy patients enrolled.

## Conclusions

The use of NSAIDs after osteotomy surgery did not negatively affect the bone healing and resulted in a decrease in narcotic consumption for deformity correction patients. Patients undergoing bone lengthening using distraction osteogenesis did experience an insignificant increase in time to healing (BHI) and no significant reduction in narcotic use. Our center has continued to use the NSAID protocol consisting of normal dose NSAIDs and acetaminophen for sustained periods of time to control surgical pain. Based on our experience, we will continue to administer these medications to patients undergoing all orthopaedic surgery in an effort to reduce opioid use. A concern over possible delayed healing times in lengthening patients will encourage our center to reduce the duration of NSAID use and continue to look for other pain control modes.
